# Ileal perforation as the initial presentation of Crohn’s disease in a 20-year-old female: a case report

**DOI:** 10.1093/jscr/rjag230

**Published:** 2026-04-04

**Authors:** Faisal M Rawagah, Rahaf E Farah, Ala’ N Fari

**Affiliations:** Department of Surgery, Princess Basma Teaching Hospital, Irbid 21110, Jordan; Palestinian Ministry of Health, Jenin District, State of Palestine; Department of Surgery, Princess Basma Teaching Hospital, Irbid 21110, Jordan

**Keywords:** ileal perforation, Crohn’s, initial, case report

## Abstract

Spontaneous small bowel perforation is an uncommon initial manifestation of Crohn’s disease, particularly in young adults. We report the case of a 20-year-old woman who presented with a 24-h history of progressive abdominal pain and vomiting. Clinical assessment revealed tachycardia, abdominal distension, absent bowel sounds, and diffuse peritonitis. Laboratory evaluation showed marked leukocytosis, and computed tomography imaging demonstrated features of ileal perforation. After initial resuscitation, urgent surgical exploration identified a perforated ileal segment with associated mesenteric inflammation. Limited ileal resection with primary stapled anastomosis and a protective diverting loop ileostomy were performed. The postoperative course was uneventful, and the patient was discharged on postoperative day six. Histopathology confirmed Crohn’s disease. This case highlights that ileal perforation may be the first presentation of Crohn’s disease and underscores the importance of early recognition and timely surgical management in acute abdomen presentations.

## Introduction

Crohn’s disease (CD) is a chronic, relapsing inflammatory bowel disorder that can affect any part of the gastrointestinal tract and is characterized by transmural inflammation, fistula formation, and stricturing complications. Although abdominal pain, diarrhea, and weight loss are the most common initial manifestations, approximately 10%–30% of patients first present with acute surgical complications, including obstruction, abscess formation, or perforation [1, 2]. Spontaneous free perforation, however, remains a rare presentation, occurring in only 1%–3% of patients with CD, and is most frequently localized to the distal ileum [3, 4].

Because free perforation often presents with sudden peritonitis and hemodynamic instability, timely diagnosis and operative management are essential. The condition may occur even in individuals without a prior diagnosis of inflammatory bowel disease, making early recognition challenging for clinicians. We present a case of spontaneous ileal perforation as the initial presentation of CD in a young female an uncommon yet life-threatening surgical emergency.

## Case presentation

A 20-year-old female with a history of peptic ulcer disease (PUD), previously treated with triple therapy, presented to the Emergency Department with a 24-h history of severe abdominal pain and repeated vomiting. The pain began in the epigastrium, progressively intensified, and became diffuse, forcing her to stop daily activities. She reported four episodes of non-bilious vomiting and denied fever, diarrhea, constipation, abdominal distention, melena, or hematochezia. She had no prior similar episodes and no weight loss. Her social history was unremarkable, and her family history included a third-degree relative with gluten-related disorders.

On arrival, she appeared ill, mildly dehydrated, and anxious. Vital signs revealed a temperature of 37.9°C, tachycardia at 123 bpm, blood pressure 103/73 mmHg, respiratory rate 28/min, and oxygen saturation 98% on room air. Abdominal examination showed distention, absent bowel sounds, and diffuse tenderness with rebound, but no rigidity or focal guarding. Rectal examination revealed heme-negative stool.

Initial investigations showed leukocytosis (WBC 29.5 × 10^3^/μl) and thrombocytosis (500 × 10^3^/μl). Kidney and liver function tests were within acceptable limits aside from a bilirubin of 12.6 μmol/l (direct 5.9 μmol/l). Electrolytes were normal, and serum amylase was mildly elevated (160 U/l). Urinalysis demonstrated mild proteinuria and glucosuria.

Resuscitation was initiated with intravenous fluids, urinary catheterization, and nasogastric decompression, yielding 500 ml of bilious fluid. Following 2 l of IV fluids, her heart rate improved from 120 to approximately 95 bpm.

**Figure 1:**
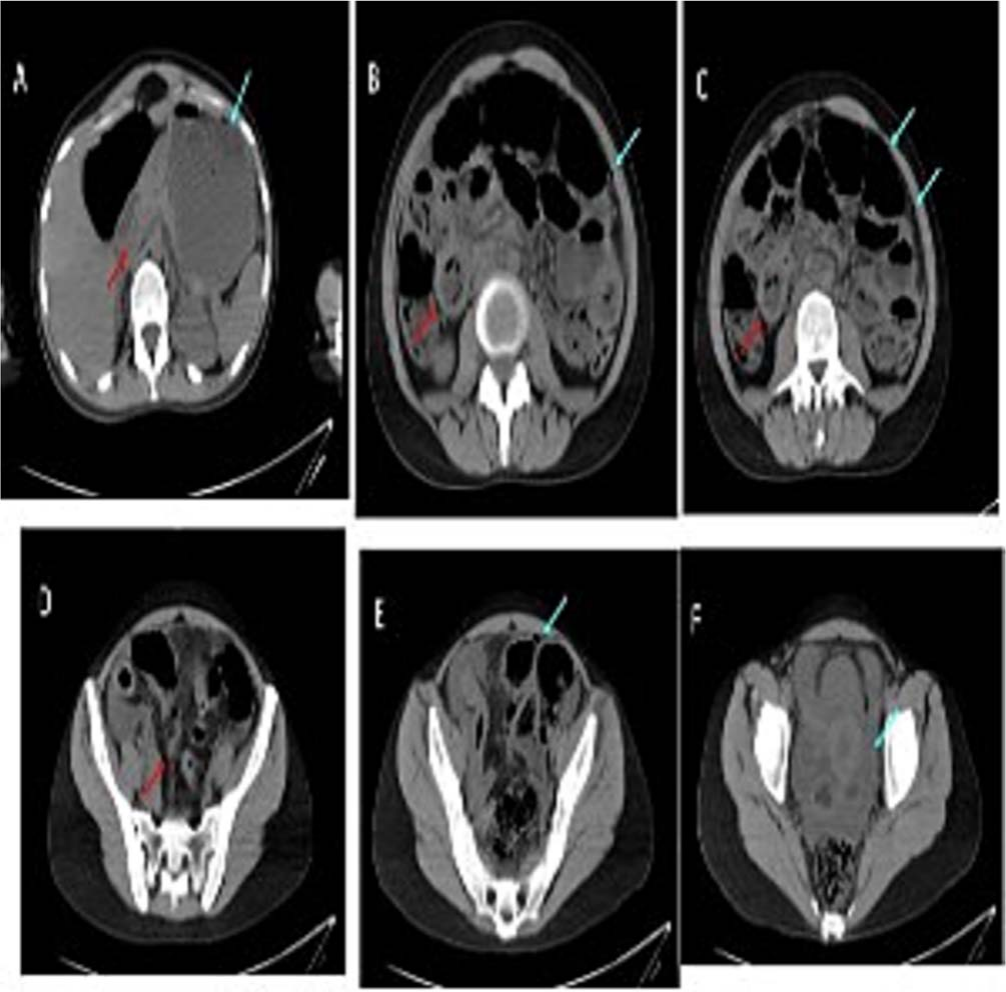
Serial images from the patient computed tomography abdominal scan that showed terminal ileum wall thickening contributing to luminal narrowing, along with surrounding fat stranding and mesenteric congestion which is associated with some adjacent prominent lymph nodes. There is few foci of extraluminal free gas reflecting bowel perforation, in addition to free fluid in the pelvis and paracolic gutters. Despite the presence of some bowel dilatation no complete bowel obstruction and the rectum lumen is preserved.

**Figure 2:**
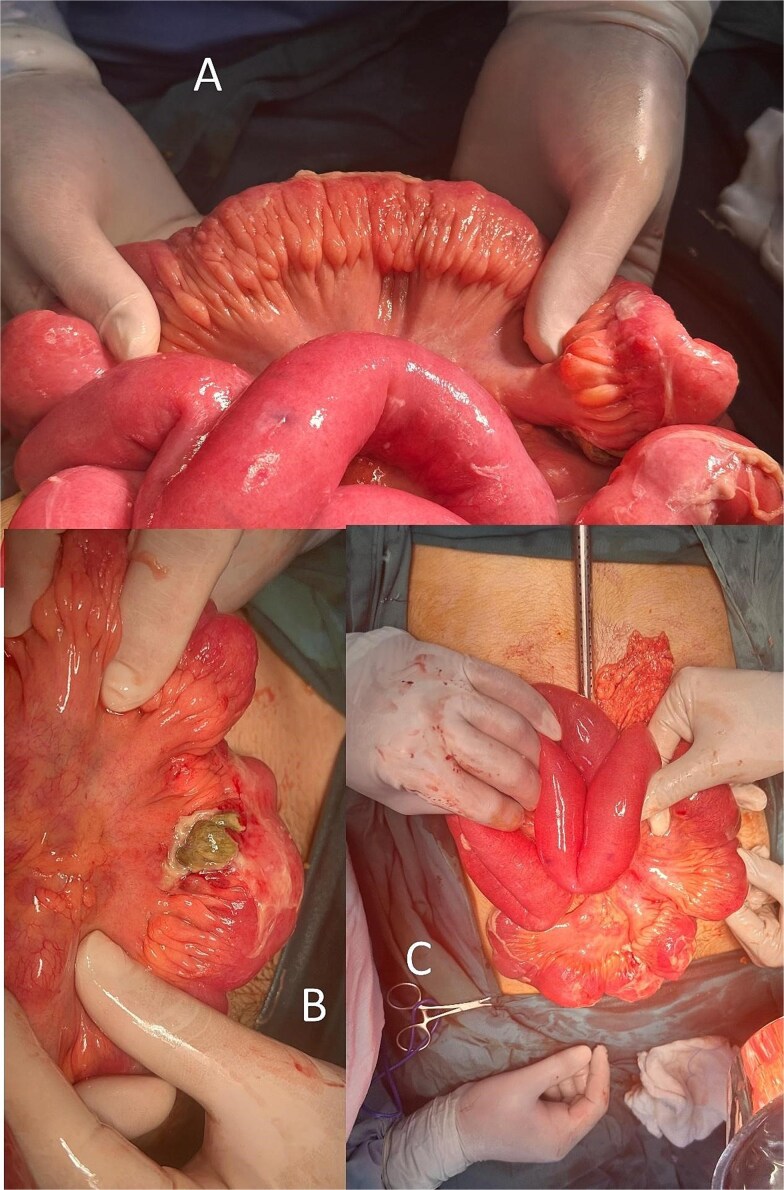
Fat creeping, ileal perforation, lymph nodes (mesenteric lymphadenitis).

Cross-sectional imaging of the abdomen demonstrated findings concerning for small bowel perforation (Fig. 1). Given the clinical and radiologic evidence of peritonitis, urgent surgical exploration was performed. Intra-operatively, a perforated segment of distal ileum with adjacent inflamed mesentery was identified (Fig. 2). Limited resection of the affected ileal segment, including associated mesentery and regional lymph nodes, was undertaken. A functional side-to-side stapled anastomosis was created, and a proximal diverting loop ileostomy was fashioned to reduce the risk of anastomotic complications.

The patient was admitted post-operatively to the ICU. She remained hemodynamically stable and afebrile. On postoperative day (POD) 1, she had 150 ml of serosanguineous drain output and 500 ml NG drainage; laboratory values were within normal limits. The ileostomy had not yet begun functioning. On POD 2, the ileostomy produced 400 ml output, the drain output decreased to <50 ml, and the NG tube and Foley catheter were removed. She developed mild hypokalemia (3.2 mmol/l), which was corrected. By POD 3, she was transferred to the surgical ward with improving abdominal distension and increasing ileostomy output (1400 ml/day). Oral intake was gradually advanced, and electrolyte supplementation provided.

She received multidisciplinary care, including gastroenterology consultation due to suspicion of CD. She was trained in stoma care and tolerated a high-protein diet. She was discharged home on POD 6 in stable condition. Histopathology of the resected ileal segment was pending at discharge and confirmed to represent transmural inflammation with surface ulceration and serous it is, bowel mucosa shows ischemic changes with congestion, edema and surface sloughing, severe lymph nodes were harvested and all of them show reactive changes and negative for malignancy. Long-term management was planned to include initiation of biologic therapy, surveillance ileoscopy through the stoma at 3 months, loopogram at 6–8 weeks, and eventual stoma reversal once disease activity is adequately controlled.

## Discussion

Spontaneous free perforation in CD is a rare and life-threatening complication, occurring in approximately 1% to 3% of patients, often as the initial manifestation of the disease [1]. While CD is characterized by transmural inflammation, free perforation into the peritoneal cavity is significantly less common than contained micro-perforations or fistulizing disease [2]. This case is particularly significant as the perforation served as the de novo clinical presentation, initially confounded by a documented history of PUD [3]. The diagnostic challenge was exacerbated by the epigastric localization of pain and non-bilious vomiting, which clinically mimicked a perforated gastroduodenal ulcer [1, 3].

The pathophysiology of free perforation in CD typically involves two distinct mechanisms: a “blow-out” proximal to a distal fibrotic stricture due to increased intraluminal pressure, or primary transmural necrosis in an acutely inflamed non-obstructed segment [4]. In this patient, the absence of prior obstructive symptoms and the intraoperative identification of a focal ileal defect suggests an aggressive, primary penetrating phenotype, classified as Montreal B3 [5]. The presence of marked leukocytosis (29.5 times 10^3^ μl) and systemic tachycardia served as critical markers of a severe inflammatory response, necessitating immediate surgical intervention rather than prolonged conservative management [2, 6].

Surgically, the primary objective in perforated CD is source control while adhering to the principle of “bowel-sparing” resection [7]. While primary anastomosis is generally favored in elective settings, the presence of fecal peritonitis and hemodynamic instability increases the risk of anastomotic dehiscence exponentially [7, 8]. In this instance, the decision to perform a limited ileal resection followed by a functional side-to-side stapled anastomosis with a proximal diverting loop ileostomy was a strategic “safety-first” maneuver [8]. This approach is supported by evidence suggesting that fecal diversion significantly mitigates the morbidity associated with potential leaks in patients with undiagnosed, active inflammation, and compromised nutritional status [6, 8].

The postoperative management of de novo CD requires a transition to a multidisciplinary framework involving gastroenterological expertise [9]. Following the histopathological confirmation of transmural inflammation and non-caseating granulomas, the focus must shift toward the prevention of early endoscopic recurrence [5, 9]. Current guidelines emphasize that early initiation of biologic therapy, specifically anti-TNF agents, is the most effective strategy to maintain remission following emergency resection [9, 10]. Our long-term protocol, including surveillance ileoscopy through the stoma at 3 months, is designed to confirm mucosal healing prior to the restoration of bowel continuity [7, 10].

## Conclusion

Spontaneous small bowel perforation can be a rare initial presentation of CD in young adults. Early recognition and prompt surgical intervention with resection and temporary diversion can be life-saving and facilitate recovery. Multidisciplinary care, including postoperative gastroenterology management and stoma education, is essential for optimizing long-term outcomes. Clinicians should maintain a high index of suspicion for underlying inflammatory bowel disease in similar acute presentations, even in patients without prior chronic symptoms.
